# Vaccine adjuvants to engage the cross-presentation pathway

**DOI:** 10.3389/fimmu.2022.940047

**Published:** 2022-08-01

**Authors:** Woojong Lee, M. Suresh

**Affiliations:** Department of Pathobiological Sciences, University of Wisconsin-Madison, Madison, WI, United States

**Keywords:** adjuvants, cross-presentation, immunity, memory, CD8 T cells, vaccines

## Abstract

Adjuvants are indispensable components of vaccines for stimulating optimal immune responses to non-replicating, inactivated and subunit antigens. Eliciting balanced humoral and T cell-mediated immunity is paramount to defend against diseases caused by complex intracellular pathogens, such as tuberculosis, malaria, and AIDS. However, currently used vaccines elicit strong antibody responses, but poorly stimulate CD8 cytotoxic T lymphocyte (CTL) responses. To elicit potent CTL memory, vaccines need to engage the cross-presentation pathway, and this requirement has been a crucial bottleneck in the development of subunit vaccines that engender effective T cell immunity. In this review, we focus on recent insights into DC cross-presentation and the extent to which clinically relevant vaccine adjuvants, such as aluminum-based nanoparticles, water-in oil emulsion (MF59) adjuvants, saponin-based adjuvants, and Toll-like receptor (TLR) ligands modulate DC cross-presentation efficiency. Further, we discuss the feasibility of using carbomer-based adjuvants as next generation of adjuvant platforms to elicit balanced antibody- and T-cell based immunity. Understanding of the molecular mechanism of DC cross-presentation and the mode of action of adjuvants will pave the way for rational design of vaccines for infectious diseases and cancer that require balanced antibody- and T cell-based immunity.

## Introduction

Although highly purified recombinant subunits from pathogens are safe, they are poorly immunogenic due to their inability to replicate, engage multiple pathways of innate immune signaling and persist, which necessitates the use of adjuvants to augment immunogenicity and program durable immunity (1–4). Protection afforded by most effective vaccines heavily relies on elicitation of neutralizing antibodies and currently used adjuvants are less effective in inducing strong CD4 and CD8 T cell-based immunity (5). For diseases that require both neutralizing antibodies and T cell immunity, such as AIDS, tuberculosis, and malaria, it will be crucial to incorporate immune adjuvants that also provoke potent T-cell immunity (6–8). To trigger robust CD8 T cell immunity by vaccines consisting of subunit antigens, it is necessary to engage the antigen processing pathway of cross-presenting dendritic cells. Currently, very few adjuvants used in licensed vaccines in the United States are known to elicit potent CTL responses.

Antigen presentation is a key regulatory process and presents a target mechanism for potentiating adaptive immune responses against extracellular and intracellular pathogens. During an immune response, antigen-presenting cells (APCs) endocytose foreign antigens, process, digest them, and load digested peptides derived from antigens to either major histocompatibility complex (MHC) class I or class II molecules (9–11). Typically, internalized antigens are degraded by endolysosomal compartments and peptides are loaded on to MHC II molecules for presentation to CD4 T cells *via* endocytic pathway. Based on this paradigm, exogenous antigens and antigens derived from pathogens that replicate in the endosomes are processed in the lysosomes and presented to CD4 T cells. By contrast, cytosolic antigens that are typically derived from endogenous sources are processed by cytosolic proteasomes and antigenic peptides are loaded on to MHC I molecules. Thus, depending upon the subcellular localization of antigens (i.e. cytosolic versus endocytic compartment), antigens are routed to two distinct protein processing pathways for loading peptides onto MHC-I or MHC-II molecules. There are exceptions to the aforementioned paradigm, because DCs in particular can process and present internalized antigens to both CD4 and CD8 T cells by cross-presentation. This process is vital because it allows the initiation of CTL immunity when DCs are not directly infected with cytosolic intracellular pathogens, such as some viruses (12–14). Significant to vaccine development, DC cross-presentation is also critical to stimulate cytotoxic T-cell mediated immunity by subunit vaccines. Hence, understanding the molecular mechanisms of DC cross-presentation is important because it stimulates an important facet of immune defense against intracellular pathogens that are not effectively controlled by antibodies or evade recognition by antibodies. In this review, we will focus on recent insights into molecular mechanisms of cross-presentation of exogenous antigens and the mechanism of action of clinically relevant vaccine adjuvants that are known to stimulate CTL immunity.

## Cross-presenting DC subsets

DC subsets can be categorized into subpopulations based on their ontogenies, gene signatures, and functions (15, 16). Several murine and human DC subsets are capable of cross-presenting exogenous antigens, but only some can efficiently cross-prime CD8 T cells. Among them, Conventional DCs (cDCs) possess all the necessary attributes for efficient cross-presentation. In mice, cDCs are broadly classified as migratory and lymphoid-resident DCs. Migratory conventional DCs are localized in non-lymphoid tissues and categorized into CD103^−^CD11b^+^ or CD103^+^CD11b^−^ subsets. Migratory cDCs internalize antigens and migrate to the draining lymph nodes for cross-presentation to T cells. On the other hand, lymphoid organs, such as lymph nodes and spleen harbor CD8a^+^ CD11b^−^ or CD8^−^ CD11b^+^ resident cDCs. In both mice and humans, cDCs can be classified as cDC1 and cDC2. The cDC1 subset is present in both lymphoid and non-lymphoid tissues and expresses high levels of a class of chemokine receptors termed X-C Motif Chemokine Receptor 1 (XCR1) (17–19). Upon antigen recognition in the context of cDC1, CD8 T cells secrete copious amounts of XC-chemokine ligand 1 (XCL1) that facilitates differentiation of effector cytotoxic T cells (20). cDCs also express high levels of RAC2, a GTPase that facilitates the assembly of NOX2 complex in the phagosomes, which in turn leads to high intra-phagosomal ROS and alkalinization of phagosomes and delayed antigen degradation within phagosomal compartments (21–23). Injection of exogenous antigen such as horse cytochrome c selectively induces apoptosis in CD8a^+^ cDCs, suggesting that CD8a^+^ cDCs are proficient in transferring exogenous antigens into cytosol compared to other DC subsets (24). Unlike other DC subsets, cDC1 also express high levels of genes that are critical for MHC-I pathway (25). Further, mice deficient in WDFY Family Member 4 (WDFY4) or Basic Leucine Zipper ATF-Like Transcription Factor 3 (BATF3) exhibit defects in their ability to prime virus-specific CD8+ T cells *in vivo* or to induce tumor rejection, suggesting that cDC1 is the major cross-presenting DC subset in mice *in vivo* (26, 27). The cDC2 subset expresses same levels of CD11c and MHC class II compared to cDC1. However, cDC2s express generally higher levels of CD4, CD11b and Sirpα, but they do not express cDC1 markers, such as DNGR-1, XCR1, and CD8α (28, 29). The homeostasis of cDC2 is also dependent on the transcription factor called IRF4, rather than Batf3 (30, 31). In terms of functions, cDC2 is important for presenting soluble antigens to CD4+ T cells and initiating TH_2_ immune responses to allergens and extracellular pathogens, as well as inducing ILC3 and TH_17_ immune responses. In sum, murine cDC1 possesses superior capacities to cross-present exogenous antigens to naïve CD8 T cells, compared to other DC subsets.

Extensive review of literature on cross-presentation by different human DC subsets has been reported elsewhere (32). Briefly, in humans, DC subsets are categorized as Blood DC antigen 1 (BDCA1)^+^ DCs (CD1c^+^ DCs), BDCA3^+^ DCs (CD141^+^ DCs), monocyte-derived DCs (moDCs), and plasmacytoid DCs (pDCs). The BDCA1^+^ and BDCA3^+^ subsets are thought to be the human counterparts of murine CD8α^−^ (CD11b^+^) and CD8α^+^ DCs, respectively. While it is well established that murine cDC1 is superior to cDC2 in terms of their ability to cross-present exogenous antigens both *in vivo* and *in vitro*, whether or how different human DC subsets mediate DC cross-presentation remains elusive. For example, all DC subsets, including cDC1 (BDCA3+), cDC2 (BDCA1+), pDCs, and moDCs are capable of cross-presenting exogenous antigens (33–38). In contrast to murine CD11b^+^ CD8a^-^DCs, BDCA1+ DCs can cross-present all sources of extracellular antigens, including soluble antigens, cell-associated antigens, and peptides (36, 38). Importantly, addition of saponin adjuvants or TLR ligands strongly augmented DC cross-presentation in BDCA1^+^ DCs (34, 39). Additionally, studies with *in vitro* differentiated (with GM-CSF and IL-4) human mo-DCs report variable results regarding their ability to effectively cross-present exogenous antigens (40–42). For example, cell-associated viral antigens could be effectively cross-presented by human mo-DCs in most studies. However, their ability to cross-present soluble proteins varied depending on the source of soluble proteins, adjuvant formulations and maturation stimuli *in vitro*. Further, Tang-Huau et al. reported that both *in vitro-* and *in vivo-*generated human moDCs cross-present using a vacuolar pathway (43). However, only ascites mo-DCs provide co-stimulatory signals to induce effector cytotoxic CD8+ T cells. Therefore, different immune subsets, especially in humans, are likely to engage different routes of DC cross-presentation, which can be harnessed by therapeutic vaccinations, in conditions, such as cancer. Future studies are warranted to investigate mechanisms to engage DC subsets to augment DC cross-presentation following prophylactic or therapeutic vaccinations.

## Molecular mechanisms of dendritic cell cross-presentation

There are excellent reviews focused on mechanisms of cross-presentation (9, 44, 45). Hence, in this review, we will focus on major concepts of DC cross-presentation that is relevant to mechanism of action of vaccine adjuvants.

### a) DC cross-presentation: vacuolar pathway

**Figure 1:**
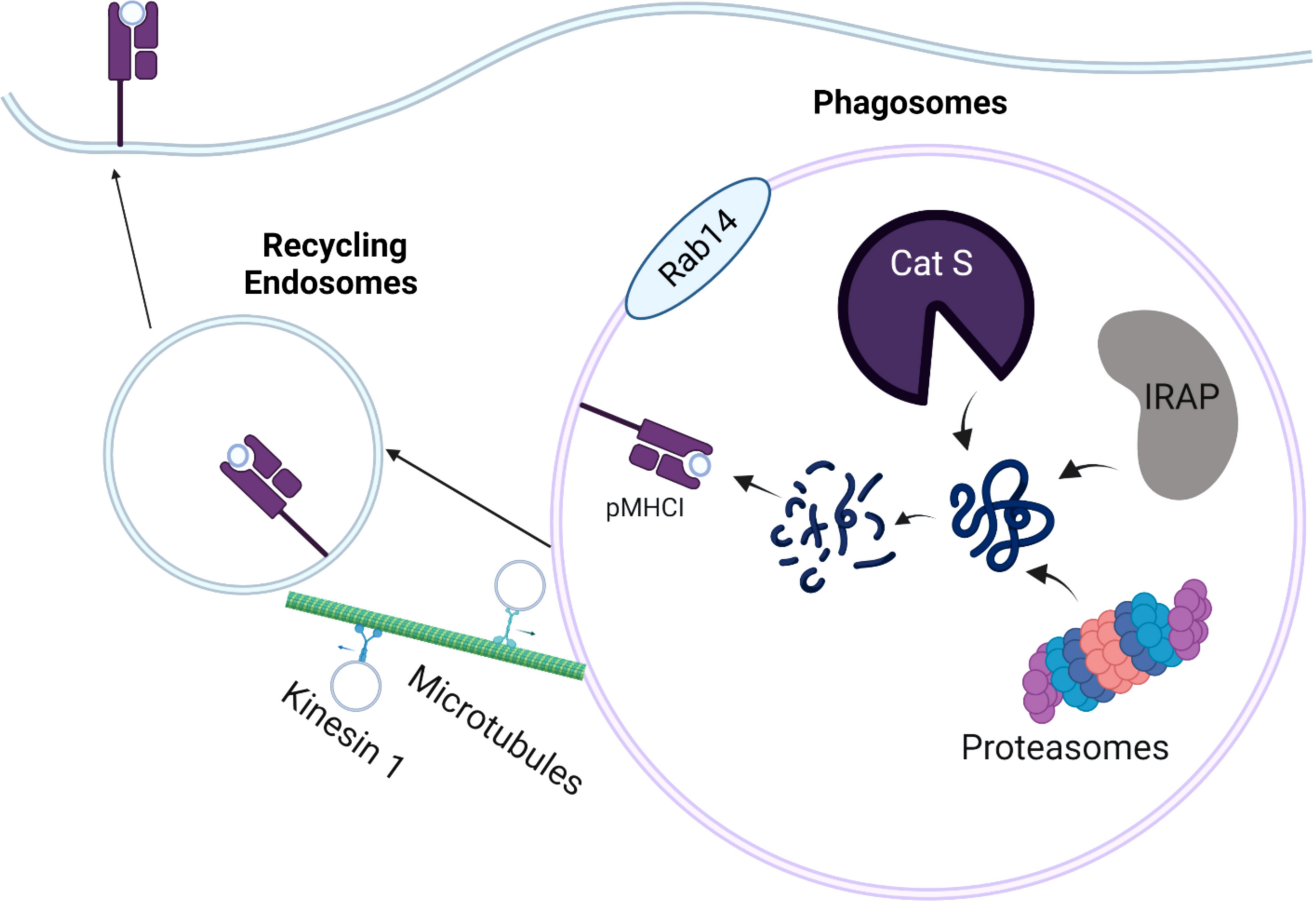
Schematic Overview of the Vacuolar Pathway of Cross-presentation. The internalized antigens are processed by residential cathepsin (cathepsin S; Cat S), Insulin regulated aminopeptidase (IRAP), or proteasomes in the Rab14^+^ and IRAP^+^ phagosomes. The processed peptides are loaded onto MHC-I molecules derived from cellular membrane. The peptide-MHC I complexes (pMHC-I) are trafficked back to the plasma membrane by recycling endosomes, which are mediated by kinesin-1 and microtubules.

Depending on the intracellular localization of antigens, cross-presentation can occur by either vacuolar pathway or cytosolic pathway. In the vacuolar pathway, exogenous antigens are endocytosed, retained in the phagosomal compartment, and further digested and trimmed by residential cysteine protease cathepsins, such as Cathepsin S (**Figure 1** **)** (46, 47). Similar to action of ERAP1 and ERAP2 in conventional MHC-I antigen presentation, endosome-localized insulin regulated aminopeptidase (IRAP) is recruited to the phagosomes to facilitate the formation of MHC-I complexes by digesting antigenic peptides generated within the endocytic (IRAP^+^, Rab14^+^) compartment (48, 49). In the absence of IRAP, phagosomal maturation is accelerated, leading to more degradative and microbicidal phagosomes (50). While it is widely believed that cytosolic proteasome is a key component in cytosolic pathway, recent work suggests that active proteasomes within cross-presenting cell phagosomes can generate intraphagosomal proteasome-generated peptides using TAP-independent mechanism (51). Hence, antigenic peptides derived from exogenous antigens can be directly digested by active proteasomes in early phagosomes, in addition to residential cathepsins and aminopeptidases within phagosomes. Once antigenic peptides are generated, the peptides are loaded onto MHC I originated from recycling endosomes, leading to the activation of CD8 T cells. In a related study, Stahl et al. linked Polylactide-co-glycolide (PLGA) and polyethylene glycol (PEG) and exogenous antigen (OVA) with substrates of cathepsin S. This study found that PLGA-PEG-OVA linked with cathepsin S substrates resulted in enhanced cross-presentation mediated by residential cathepsins in DCs (52). However, such delivery system also appears to engage cytosolic pathway, as further evidenced by enzyme-triggered antigen release from endosomes into cytosol.

To gain better insight into the dynamics of recycling endosomes, Belabed et al. examined whether a motor protein, kinesin-1 promoted antigen cross-presentation through the scission of tubulations from early endosomes in DCs (53). In the absence of kinensin-1, antigen degradation, the downregulation of endosomal pH, and MHC-I recycling were severely impaired in DCs, resulting in reduced DC cross-presentation. This suggests that kinesin-1 may act as a vital checkpoint that controls antigen degradation, MHC-I recycling, and endosomal pH for optimal DC cross-presentation by the vacuolar pathway.

### b) DC cross-presentation: cytosolic pathway

**Figure 2:**
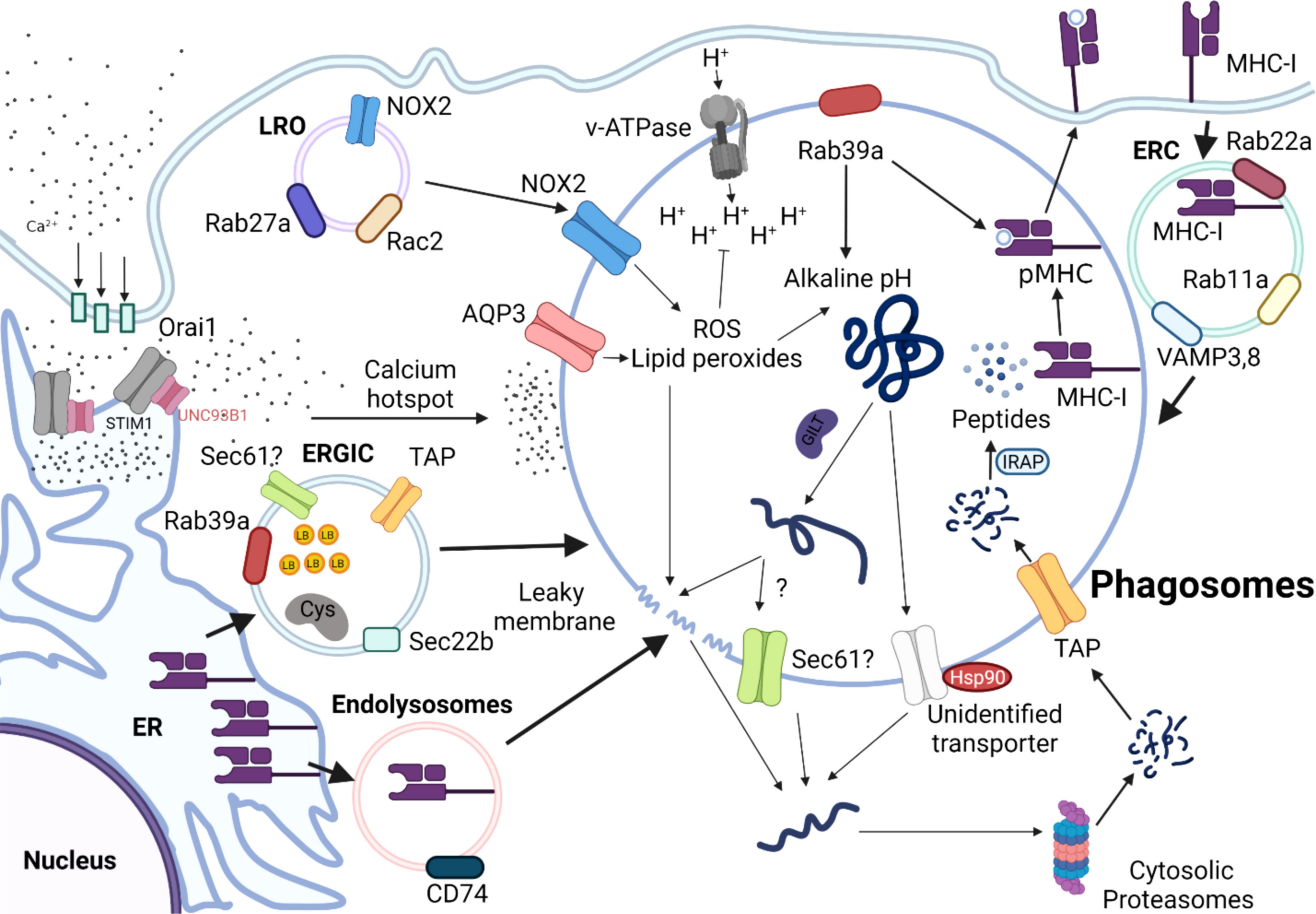
Schematic Overview of the Cytosolic Pathway of Cross-presentation. Antigens are internalized into the alkaline phagosomes by a process that is tightly regulated by various proteins, such as NADPH oxidases 2 (NOX2), Aquaporin 3(AQP3), and Rab39a. NOX2 complexes are recruited to the phagosomes by lysosome-related organelle (LRO) mediated by Rab27a and Rac Family Small GTPase 2 (Rac2). ER-Golgi intermediate compartment (ERGIC) derived from endoplasmic reticulum (ER) deliver various intracellular components required for cytosolic pathway, such as Rab39a, Transporter Associated With Antigen Processing (TAP), and SEC22 Homolog B 61(Sec61), which is coordinated by sec22b. The antigens are unfolded by Gamma-interferon-inducible lysosomal thiol reductase (GILT), which will be released into cytosol, presumably by leaky membranes effected by sec61, or heat shock protein (HSP90). Partially unfolded peptides will be further processed by cytosolic proteasomes and transported back to the phagosomes by TAP. The antigenic peptides are processed by IRAP, so that they can be readily loaded onto MHC-I molecules to form pMHC-I molecules are either derived from endolysosomes derived from ER, which is mediated by CD74, or recycled from endosomal recycling component (ERC) from plasma membrane.

In the cytosolic pathway, exogenous antigens are first endocytosed into phagosomes, translocated to the cytoplasm, and subsequently degraded by cytosolic proteasomes (**Figure 2** **)**. First, lysosome-related organelles (LRO) promote the delivery of NADPH oxidase complex (NOX2) to phagosomes by a process mediated by Rab27a and Rac2 (21, 54, 55). NOX2 complex in the phagosomes generates free radicals (reactive oxygen species, ROS), thus increasing pH, which in turn contributes to the alkaline and less proteolytic environment within phagosomes (56). Particularly, maintenance of alkaline phagosome environment is critical for delaying antigen degradation and improving the efficiency of DC cross-presentation. The partially digested antigens are subsequently translocated from phagosomes into cytosolic compartment for further processing by cytosolic proteasomes.

Excessive degradation of antigens mediated by lysosomal proteases negatively affects the efficiency of cross-presentation to CD8 T cells but promotes MHC-II-presentation to CD4 T cells (57, 58). Consistent with this idea, Samie et al. showed that prolonged stimulation with LPS elevates expression of the transcription factor EB (TFEB), which triggers lysosomal maturation and activation of lysosomal proteases (59). Later studies by Bretou et al. have mechanistically dissected the regulatory role of TFEB-TRPML1 (Transient Receptor Potential Mucolipin 1) axis in lysosomal calcium channels, DC migration and motility *in vivo* (60). The p38α signaling pathway in DCs has essential roles, as it tightly regulates DC maturation and production of pro-inflammatory cytokines, including IL-6, IL-12, and TNF-α (61–63). In accordance with this concept, Zhou et al. found that deletion of p38α in cDCs resulted in impaired cross-presentation, thereby facilitating antigen degradation, and reducing the production of IL-12p40 and IL-12p70 (64). As lysosomal acidity increases in DCs, the efficiency of cross-presentation wanes due to the premature state of phagosomes. In particular, p38 likely acts as a negative regulator of TFEB-mediated lysosomal biogenesis, especially in microglia (65–67). Hence, careful modulation of lysosomal activity mediated by p38-TFEB signaling axis is also likely critical in determining the fate of an internalized antigen and in regulating the balance between the two exogenous antigen-presenting pathways in DCs.

How antigens trapped in the endosomes gain access to the cytosol remains controversial, but it is widely believed that translocation of antigens from phagosomes into cytosol can occur *via* 1) translocon/transporter proteins, or 2) chemical-induced membrane damage. For transporter-mediated antigen leakage, disulfide bonds of antigens are reduced and unfolded by GILT (Gamma-interferon-19 inducible lysosomal thiol reductase) or Hsp90, respectively (68, 69). Subsequently, unfolded polypeptides can escape from phagosomes into cytosol by ER-associated degradation (ERAD) member, sec61, which are refolded by Hsp90. To test that Sec61 is vital for endosomal leakage of antigen, using a Sec61-specific intracellular antibody, Zehner et al. trapped Sec61 in the ER and prevented its transport to endosomes, thereby blocking antigen translocation and cross-presentation (70). Expression of ER intrabody inhibited antigen translocation and cross-presentation, which demonstrated that endosomal Sec61 mediates antigen transport across endosomal membranes. Moreover, the authors showed that the recruitment of Sec61 to endosomes, and hence antigen translocation and cross-presentation are dependent on DCs’ activation by Toll-like receptor (TLR) ligands. However, Grotzke et al. recently reported that a chemical inhibitor of Sec61, mycolactone, did not influence antigen dislocation from the cytosol, but rather severely inhibited protein import into the ER (71). While both studies have shown that sec61 blockade negatively affects DC cross-presentation, Sec61’s role in regulating antigen leakage is likely more complex.

By contrast, for chemical-induced membrane damage, ROS and lipid peroxides create leaky membranes in the phagosomes. Using elegant sets of translocation assays with various model antigens, Dingjan et al. demonstrated that pharmacological inhibition or genetic knockdown of NOX2 reduced intracellular levels of lipid peroxides, leading to reduced leakage of antigen from endosomes and dampened cross-presentation (72). A later study also found that VAMP8-mediated NOX2 recruitment to endosomes is necessary for antigen release from phagosomes (73). Alteration of lipid structure presumably disrupts endosomal membrane integrity, thereby facilitating antigen translocation to the cytoplasm. Nalle et al. demonstrated that hydrogen peroxide-transporting channel aquaporin-3 (AQP3) is essential for H_2_O_2_ entry into the endosomes; subsequently H_2_O_2_ affects lipid peroxidation and endosomal antigen leakage (74). This, in turn, leads to phagosomal membrane rupture, releasing antigens into the cytoplasm that can be cross-presented by MHC-I. In recent research that examined the physiological relevance of antigen leakage mediated by ROS *in vivo*, stimulation of DCs by complement protein C5a augmented ROS-mediated antigen leakage resulting in efficient DC cross-presentation in the Peyer’s patches (75). In the same study, the authors further demonstrated that CD8 T cell immunity engendered by C5a signaling provides protective immunity against oral *Listeria* infection. Hence, C5a signaling appears to play a predominant role in eliciting DC cross-presentation thereby augmenting ROS production *in vivo*, at least in the context of intestinal pathogens.

Moreover, the polymer polyethyleneimine (PEI)-based platform has been used to exploit the proton sponge mechanism to induce osmotic imbalance within phagosomes. Osmotic imbalance triggers rupture of the antigen-containing phagosomes, and antigen leakage into the cytoplasm (76, 77). High-throughput screening of small molecules recently identified two compounds (prazosin and tamoxifen) that can increase endosome-to-cytosol import, which enhanced anti-tumor immunity (78). Another study suggested that Alum-linked antigen augmented cross-presentation stimulated potent effector T cell responses, boosted tumor-infiltrating lymphocytes, and decreased the Treg/CD8 ratio (79). Hence, therapeutic development of small molecules or chemical modification of the antigen that can harness endosome-to-cytoplasm import may be used as an immunotherapeutic strategy to enhance CD8 T cell immunity through augmenting DC cross-presentation. More details on antigen export to the cytosol during cross-presentation are extensively reviewed elsewhere (80), but in summary, conventional antigen leakage from endosomes can be accomplished by transporter proteins or chemical-induced membrane damage.

As phagosomes undergo maturation, they directly interact with ER at their membranes to exchange various molecules that promote their maturation (81). Recruitment of specific sets of ER and ER-Golgi intermediate compartment (ERGIC) components to phagosomes is mediated by the ER-resident SNARE Sec22b. ERGIC may contain protease inhibitors, such as Cystatin C and lipid bodies (LB), which can directly regulate phagosomal acidity and proteolytic activity in phagosomes (82, 83). Knock-down of Sec22b in BMDCs by shRNA dampens antigen leakage from endosomes into the cytosol. The follow-up study also generated a conditional DC-specific mutation in the *sec22b* gene and further interrogated the intrinsic role of sec22b in DCs. In this study, the authors discovered that sec22b is vital for eliciting CD8 T cell responses to dead cells and for eliciting effective anti-tumor immune responses during anti–PD-1 treatment in mice (84). However, Wu et al. reported that sec22b plays a dispensable role in cross-presenting antigens both *in vivo* and *in vitro* (85). Notably, reduced DC cross-presentation was attributed to off-target effects of the shRNA. Interestingly, recent work by Rock group suggests that Rab39a acts as a regulatory transport protein, and promotes the recruitment of NOX2 complex and sec22 on mature phagosomes, leading to an increase in ROS in the phagosomes (86). This, in turn, results in phagosome alkalinization and delaying antigen processing. Rab39a also facilitates the generation of antigenic peptide-loaded MHC-I complexes in phagosomes. Therefore, in conjunction with Rab39a, sec22b appears to play a vital role in shuttling different cellular components required for cytosolic pathway from ER to the phagosomes.

Calcium signaling is linked to various DC effector functions, such as phagocytic capacities, maturation, and migration (87, 88), but the exact regulatory immune checkpoint that governs calcium signaling and cross-presentation has not been well established. Nunes-Hasler et al. identified STIM1 (store-operated-Ca2+-entry regulator) as an important checkpoint for effective DC cross-presentation (89). The absence of STIM1 reduced the efficiency of DC cross-presentation by impairing phagosomal proteolysis, IRAP recruitment, and fusion of phagosomes. This suggests that the delivery of endolysosomal enzymes to phagosomes mediated by STIM1-dependent calcium signaling may be required for effective DC cross-presentation. Uncoordinated 93 homolog B1 (UNC93B1) has been implicated in cross-presentation; UNC93B1 is activated by TLR triggering and controls the intracellular trafficking of TLRs from the ER toward endosomes (90–92). Another report by Maschalidi et al. further showed that UNC93B1 (an ER protein crucial for regulating intracellular TLR signaling) induces STIM1 oligomerization, which facilitates calcium ion efflux from ER into cytosol to induce DC cross-presentation (93). Using UNC93B1 mutants that cannot oligomerize with STIM1, authors further demonstrated that less calcium ion influx leads to less antigen degradation and phago-lysosomal fusion. Consequently, less antigens are exported from phagosomes into cytosol, leading to deficient DC cross-presentation. At a biochemical level, Wang et al. demonstrated that UNC93B1 acts a chaperone that facilitates the formation of resting STIM1 dimers under calcium ion depletion (94). As a result, the interaction between Orai1 (Ca^2+^ channels)-STIM1 results in an influx of calcium ions from extracellular milieu into the cytoplasm.

Translocated antigens are further digested into antigenic peptides by cytosolic proteasomes. The antigenic peptides are transported into nascent phagosomes by TAP transporters, which are further trimmed by IRAP, so that the peptides are ready to be loaded onto MHC I molecules. For cytosolic pathway, the primary source of MHC-I is the endosomal recycling component (ERC) marked by Rab11a, Rab23, VAMP3, and VAMP8 (95–97). Here, MHC I from the cell surface could be recycled by ERC and transported towards cross-presenting phagosomes using endocytosis. As another source of MHC I, endolysosomes mediated by CD74, aid in loading exogenous peptides to the phagosomes (98).

Multiple studies also demonstrate that cytosolic pathway can occur independently of TAP (99–105). Using TAP-deficient DCs, Merzougui et al. showed that TAP plays a vital role in recycling MHC I molecules to the phagosomes, and antigenic peptides generated by cytosolic proteasomes are transported to the phagosomes by an alternative transporter (102). In later studies, they found that the generation of melanoma peptide (PMEL^209-217^) requires cytosolic proteasomes for cross-presentation, but not TAP or tapasin for peptide loading on MHC-I molecules (101). This could be explained by two possible mechanisms. First, not all peptides require TAP transporters for translocation to the phagosomes, but they can still be transported to the phagosomes *via* unknown energy-dependent mechanism (106). As described earlier, residential proteasomes within phagosomes can also further trim exogenous antigens into antigenic peptides, which explains why such cross-presentation can occur in the absence of TAP (51). Recently, Barbet et al. elucidated an alternative mechanism for TAP-independent cross-presentation, in which MHC-I peptide complex is recycled to ERGIC complex, rather than ERC (100). ERGIC complex loaded with MHC-I can be delivered to phagosomes independent of TLR signaling, suggesting that cross-presentation can still occur normally, even when classic MHC-I presentation and endosomal recycling compartment–dependent cross-presentation pathways are impaired.

## Regulation of DC cross-presentation by extracellular receptors

Certain extracellular receptors mediate uptake of extracellular antigens and route them into phagosomal compartments in DCs. Therefore, directly targeting antigens to specific extracellular receptors expressed by DCs, such as Fc-γ receptors, DC-SIGN, DEC-205, and XCR1, has been an effective strategy for enhancing DC cross-presentation (107–110). Among them, CLEC9a is a group V C-type lectin-like receptor that is expressed by cDC1 and used for recognizing actin filaments by dead cells to facilitate DC cross-presentation (111–113). In addition to its role in antigen uptake, Clec9a also appears to route antigens to early and recycling endosomes to enhance DC cross-presentation in cDC1 (114). Because targeting Clec9a enhances MHC I presentation in cDC1, targeting antigens to mouse Clec9A enhances CD8 T cell responses (112, 113). Additionally, Clec9A-mediated antigen delivery elicits potent humoral immune responses in mice and non-human primates, suggesting that Clec9a-based antigen delivery can induce potent, balanced humoral and cell-mediated immunity (115–117). Clec9a-based antigen delivery is shown to engender protective immunity against infections, including malaria and influenza, as well as cancer (117–119).

The regulatory mechanism of Clec9a-mediated DC cross-presentation has been explored at both cellular and systemic levels. Canton et al. identified that engagement of Clec9a triggers SYK signaling, which subsequently leads to NOX2-mediated escape of phagosomal contents into the cytosol. Hence, Clec9a could possibly act as a checkpoint of cytosolic pathway that allows leakage of exogenous antigens into cytoplasm to activate CD8 T cells. Giampazolias et al. discovered that gelsolin, an abundant actin-binding protein found in the plasma and produced by tumors, perturbs the binding between DNGR-1 and F-actin, resulting in poor interaction between dead cancer cells and cDC1 and dampened DC cross-presentation (120). Tullett et al. discovered a negative regulatory mechanism of Clec9a-mediated DC cross-presentation, where ubiquitin ligase RNF41 interacts with Clec9A at the steady state. Following uptake of dead cells, RNF41 dissociates from Clec9A to increase the levels of Clec9a to augment effective DC cross-presentation. Therefore, RNF41 acts as a negative regulator of Clec9A in the context of cross-presentation of deal cell-derived antigens (121).

Ding et al. identified a negative role of a lectin family member, Siglec-G in DC cross-presentation (122). The authors found that the expression of Siglec-G in CD8a+ DC results in poor assembly of NOX2 complexes of DC phagosomes, leading to acceleration of antigen degradation and decreased formation of MHC class I–peptide complexes. Intriguingly, Streng-Ouwehand et al. chemically modified the chicken ovalbumin by attaching Lewis^x^ (LeX) to target C-type lectin receptor called MGL1 (103) and found that such modification of OVA resulted in a potent CD8 T cell response. As a mechanism, the authors proposed that cross-presentation of LeX-modified antigens is independent of TAP1- or Cathepsin S. Intriguingly, LeX-modified antigens are routed to Rab11^+^/LAMP1^+^ compartments (recycling endosomes/lysosomes), rather than Rab11^+^/EEA1^+^ (recycling/early endosomes) compartments.

## Antigen transfer and immune cross-talks to enhance DC cross-presentation

Another alternative mechanism that can facilitate DC cross-presentation is transfer of antigenic peptides from other immune cells to DCs by direct cell-cell contact (123, 124). Sachheri et al. initially showed that exposure of melanoma cells to *Salmonella* augments the expression of a gap junction protein, Connexin 43; gap-junction mediated by Connexin 43 is critical for antigenic peptide transfer from cells infected with intracellular bacteria to DCs (125). Mazzini et al. demonstrated that CX3CR1^+^ macrophages process antigenic peptides for transfer to CD103^+^ dendritic cells. It was further confirmed that CD103^+^ cells deficient in Cx43 cannot cross-present antigens *in vivo*, which results in poor oral tolerance, an active process of immune unresponsiveness to orally ingested antigens (126). Further, Huang et al. showed that *ex vivo* antigen-loaded monocytes elicit potent therapeutic anti-tumor T cell responses in mice by promoting efficient antigen transfer mediated by gap junction *via* connexin 43 between CD8a^+^ DCs and monocytes (127). Importantly, a recent study demonstrated that Dioscin (steroid-based saponin) increases DC cross-presentation and enhances gap junctions between melanoma and DCs, culminating in greater anti-tumor killing effects of CD8 T cells (128). Taken together, these data suggest a putative role of monocytes and macrophages in transferring antigenic peptides to DCs to promote CD8 T cell responses, that can be leveraged for enhancing cell-mediated immunity.

In both humans and mice, numerous studies suggest that pDCs endocytose, process, and present exogenous antigens to CD8 T cells, presumably using cross-presentation (38, 129, 130). Oberkampf et al. reported that induction of cross-presentation by pDCs is regulated by mitochondrial ROS-dependent mechanism, including antigen degradation and phagosomal pH (131). Fu et al. demonstrated that the antigens only delivered to pDCs (anti-Siglec-H-OVA) could be cross-presented to CD8 T cells *in vivo* (132). However, pDCs alone were not sufficient to directly cross-present antigens to CD8 T cells. Rather, antigen transfer from pDC to cDC1s mediated by exosomes-derived from pDCs were critical for priming CD8 T cells. Strikingly, both pDCs and cDCs expressed MHC I-antigen complexes at their surfaces, but only cDCs, and not pDCs were able to induce robust proliferation of naïve OT-I cells *ex vivo.*

Another important immune cell that can presumably interact with DCs is the platelet, which have critical roles in hemostasis, vascular homeostasis, and immunoregulation (133–135). Consistent with these roles, Han et al. found that P-selectin from platelets interact with PSGL1 to activate peripheral blood monocytes to augment antigen cross-presentation by forming an adhesion synapse (136). The authors termed platelet-matured cells as “physiological DCs” because they were generated in the absence of exogenous cytokines. The authors also confirmed that physiological DCs mount a robust cell-mediated immunity, compared to GM-CSF-derived BMDCs. Hence, leveraging the ability of monocytes to interact with platelets can be used as a powerful strategy for physiological DC-based immunotherapies to mount cell-mediated immune responses.

## Adjuvants and cross-presentation

### A) Alum

In the 1920s, Glenny et al. observed that guinea pigs injected with an emulsion containing diphtheria toxoid precipitated with potassium alum were better protected against repeated intradermal injections with diphtheria toxin, than those injected with toxoid alone. Since then, aluminum hydroxide (Alum) had been the primary adjuvant licensed for human use for 70 years. Alum has been safely used in many human vaccines as an adjuvant for promoting antibody and TH2 immune responses; hence Alum often serves as a benchmark to evaluate novel adjuvants in vaccine research. A key mechanism proposed for Alum-mediated adjuvant effects includes an antigen depot effect, in which antigens are stored and slowly released from the sites of immunization (137, 138). When antigens are retained at the injection site, such retention can induce local NLRP3-dependent inflammasome activation, leading to prolonged inflammation accompanied by the production of IL-1β and IL-18 and a potent polarization of T_H_2 immunity and antibody responses (139–142). It is also postulated that Alum can trigger the release of danger-associated molecular patterns (DAMPs), including uric acid, alarmin and double-stranded RNA from target cell, which increase the overall immune responses to vaccines (143, 144). Aggregated alums are positively charged microgels (1–10 µm) in aqueous solutions that attach to and spread on dendritic cells (DCs) to trigger lipid sorting of the cellular membrane. As a result, exogenous antigens formulated with Alum will be internalized without being phagocytosed together with Alum. The internalized antigens formulated with Alum will be processed *via* lysosomes using conventional MHC II presentation pathway, but Alum remains very ineffective for eliciting CD8 T cell responses.

**Figure 3:**
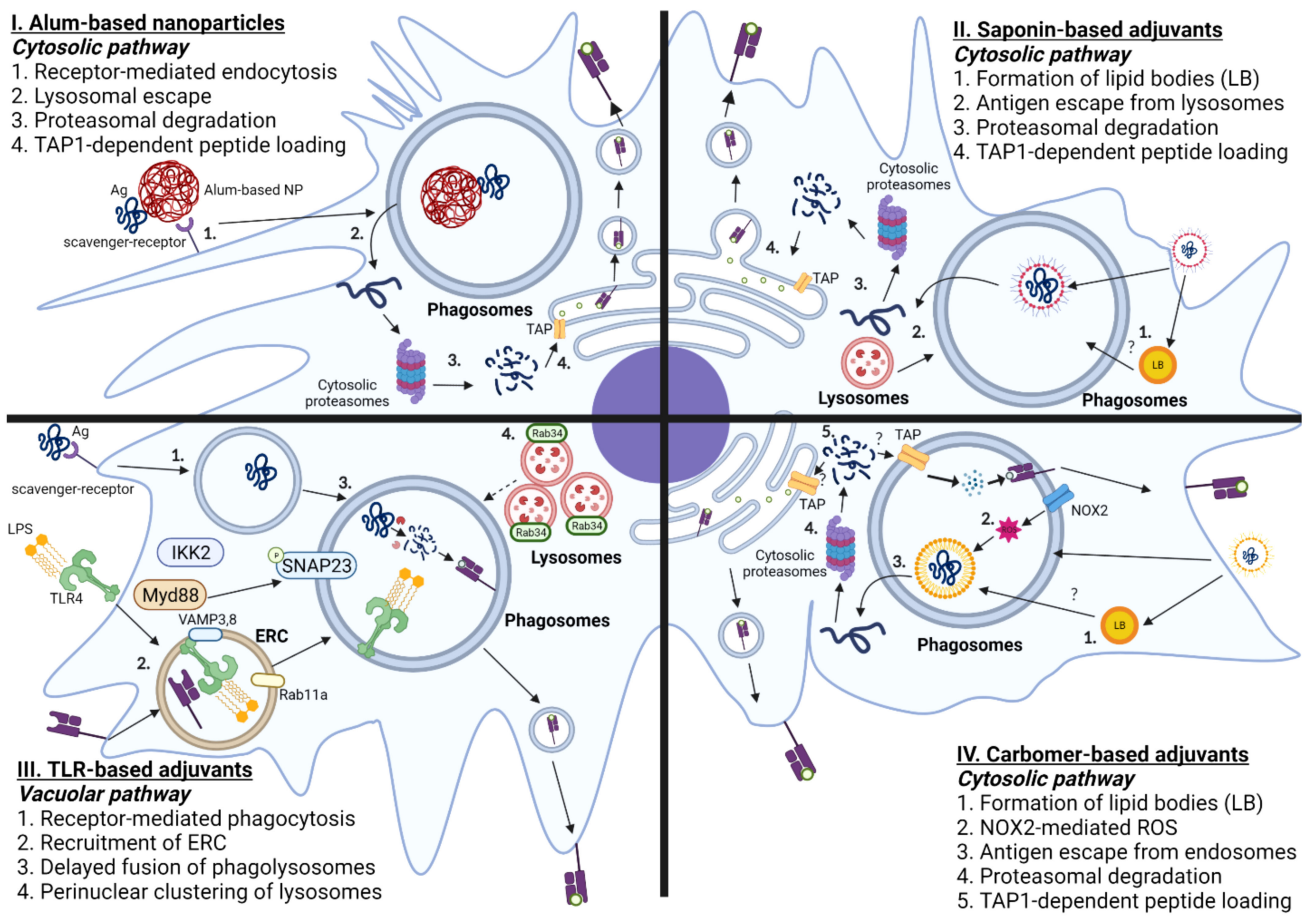
Mechanisms of Adjuvant-mediated Cross-presentation in DCs. I. Alum-based nanoparticles: Antigens that are coupled with Alum-based nanoparticles are taken up by scavenger receptor A. Antigens are translocated from phagosomes-to-cytosol, further processed by cytosolic proteasomes, and loaded onto MHC-I molecules by TAP-dependent mechanism. II. Saponin-based adjuvants: Saponin-based adjuvants induce the formation of intracellular lipid bodies (LB). Internalized antigens are localized in the phagosomes, which are released into cytosol facilitated by lysosomal proteases, and degraded by cytosolic proteasomes. The digested peptides are translocated back to ER by TAP transporters and loaded onto MHC I molecules. III. TLR-based adjuvants: Ligation of TLR agonist induces phagocytosis of antigen mediated by Myd88-IKK2-SNAP23 and recruitment of endosomal recycling complex, marked by Rab11a and VAMP 3/8. TLR-based adjuvants also elicit the formation of perinuclear clustering of lysosomes, which leads to delayed fusion of phagolysosomes. IV. Carbomer-based adjuvants: Carbomer-based adjuvants induce the formation of intracellular lipid bodies. Carbomer-based adjuvants also induce the production of ROS in the phagosomes. The internalized antigens escape from phagosomes to cytosol, which are subsequently processed by cytosolic proteasomes, translocated using TAP transporters, and loaded onto MHC-I molecules.

To overcome this caveat, several labs have attempted to optimize the biochemical and/or biophysical properties of Alum to augment DC cross presentation, as shown in **Figure 3**. An earlier report demonstrated that Alum itself taken by APCs could induce lysosomal leakage, which makes Alum an attractive candidate for engaging cytosolic pathway of DC cross-presentation to activate CD8 T cells. Li et al. demonstrated that conjugation of OVA to alpha-alumina nanoparticles resulted in efficient cross-presentation of the OVA antigen both *in vitro* and *in vivo*, by triggering autophagic flux (145). Subsequently Jiang et al. turned Alum from gel into nano-sized vaccine carriers AlO (OH)-polymer nanoparticles (APNs) loaded with CpG and examined their adjuvanticities to potentiate CD8 T cell responses. They found that APN internalization in DCs was completely reduced in the presence of dextran sulfate and fucoidan, both of which are ligands for the scavenger receptor. Mechanistically, APN-driven cross-presentation was abrogated by MG-132 (proteasome inhibitors) and brefeldin A (ER transporter inhibitors) in DCs. Additionally, APN itself was found to escape from lysosomes, which presumably promotes the access of antigens into cytosol for further processing by cytosolic proteasomes. Further, a recent study has demonstrated that intramuscular immunization of an alum-stabilized Pickering emulsion (PAPE) with RBD of spike protein showed robust IFN-γ-producing CD8 T cells in a COVID-19 vaccine (146). Ren et al. also found that coated rehydragel (aluminum hydroxide wet gel suspension) with cationic polyethyleneimine (PEI) facilitated DC cross-presentation (147). Unlike Alum-based nanoparticles, Rehydragel/PEI-mediated DC cross-presentation requires both lysosomes and proteasomes as a part of machinery to activate CD8 T cells. This suggests that rehydragel-PEI-mediated DC-cross presentation engage both vacuolar and cytosolic DC cross-presentation. In line with this conclusion, Alum-based nanoparticles coupled with CpG also engage both vacuolar and cytosolic pathways, in contrast to TLR agonists that mainly engage the vacuolar pathway and the Alum-based nanoparticles that engage the cytosolic pathway. Together, it is possible to engineer biochemical and biophysical attributes of Alum to elicit optimal CD8 T cell-based immunity by engaging the cytosolic pathway.

### B) MF59

MF59 is an adjuvant consisting of squalene (4.3%) with stabilizing non-ionic surfactants Tween 80 (0.5%) and Span 85 (0.5%) in citric acid buffer. Fluad^®^, an MF59-adjuvanted seasonal influenza vaccine, was first licensed for the elderly in 1997 and has since been approved for use in human vaccines in over 30 countries, including the United States (148). In contrast to Alum, MF59 induces a relatively balanced T_H_1/T_H_2 immune responses by forming inflammatory conditions without forming antigen depot (149–151). The mechanism of MF59 has been reviewed elsewhere (152). Briefly, MF59-adjuvanted vaccines augment the production of chemokines and inflammatory cytokines (CCL2, CCL3, CCL4, and IL-8), recruit innate immune cells, such as monocytes and neutrophils, trigger production of DAMPs such as uric acid, ATP, and induce apoptosis of innate immune cells at the injection site (148, 153–157). Recruitment and activation of innate cells, along with DAMPs, will then occur in the vaccine-draining lymph nodes to amplify humoral and cell-mediated immune responses to the vaccine antigens. Unlike Alum, MF59 does not potently induce inflammasome activity in BMDC *in vitro* (158). MF59 does not engage NLRP3, but instead requires MyD88 to enhance bactericidal antibody-based responses (158). Because MF59 does not activate TLR-dependent signaling in DCs *in vitro*, MF59-mediated adjuvanticity likely requires MyD88 for TLR-independent signaling pathways. In contrast to this, Ellebedy et al. has shown that ASC is crucial for adjuvanticity of MF59, but not NLRP3 or caspase-1 (159); this study found that antigen-specific IgG antibody responses were significantly impaired in the absence of ASC. Hence, ASC plays an indispensable role in the induction of humoral immunity that is independent of NLRP3-dependent inflammasome activation.

Kim et al. discovered that MF59 elicits potent humoral and CD8 T cell responses in mice. They also identified the RIPK3-dependent necroptotic death of lymph node resident macrophages as a key mechanism by which squalene-based adjuvants elicit CD8 T cell immunity (160). The authors showed that mice deficient in RIP3 were unable to mount antigen-specific CD8 T cell responses in the liver and lung when they are subcutaneously immunized with MF59-adjuvanted vaccines. In contrast, mice deficient in MLKL (a downstream gene of necroptosis) mounted normal antigen-specific CD8 T cell responses *in vivo*. This suggests that the adjuvant effects mediated by MF59 requires RIPK3 that is independent of necroptosis, as they do not require MLKL. Antibody responses mediated by MF59 occur normally in mice deficient in RIPK3 or caspase-1. However*, in vivo* administration of pan-caspase inhibitors abrogated antibody-based responses elicited by MF59, suggesting that apoptotic caspases are required for MF-59 mediated antibody responses. Hence, MF59 may be used as an immune adjuvant that can be incorporated to elicit both humoral and cell-mediated immunity. Future studies are warranted to examine whether MF59-based vaccines confer T cell-based protection to pathogens and how long memory CD8 T cells induced by MF59-based vaccines persist in lymphoid organs. Further, it will be important to determine whether MF59-based adjuvants engage vacuolar or cytosolic pathway of DC-cross presentation, as this has not been investigated yet.

### c) Lipid nanoparticles (LNP)-mRNA vaccines

Lipid nanoparticles (LNPs) are composed of PEGylated lipids that encapsulate mRNA as delivery vehicle, which has been successfully used for some COVID-19 vaccines (161, 162). Such delivery system increases the stability of mRNA, because mRNA can be protected from enzymatic degradation. The cellular mechanism of antigen presentation mediated by LNP-mRNA vaccines has been described extensively elsewhere (161, 163, 164). Briefly, mRNA encapsulated with LNPs can be endocytosed into the dendritic cell; mRNA will need to escape from LNP and endosome for subsequent translation of the target antigens by ER and Golgi apparatus (165). Translated proteins will be further processed by cytosolic proteasomes, loaded on ER, and presented to MHC-I molecules to potentiate both humoral and cell-mediated responses. Hence, the cellular mechanism of LNP-mRNA vaccine is similar to the processing of endogenous antigens for eliciting T cell-mediated responses.

For LNP-mRNA-based vaccines, the encapsulated mRNA itself can serve as both immunogen and adjuvant, because of intrinsic immunostimulatory properties of RNA. The current LNP-mRNA vaccines consist of purified, *in vitro*-transcribed single-stranded mRNA (ssRNA) with modified nucleotides to reduce binding to various pathogen recognition receptors, such as TLR and inflammasomes (166). However, ssRNA derived from vaccines can be recognized by endosomal TLRs (TLR3 and TLR7). Different components of inflammasome activation, including NOD2 and MDA5, can also bind to cytosolic ssRNA derived from LNP-mRNA vaccines. Consequently, binding of ssRNA derived from LNP-mRNA vaccines can lead to vaccine-induced systemic inflammation, leading to cellular recruitment of various innate and adaptive immune cells and establishment of inflammatory milieu, like other vaccine adjuvants.

### d) Saponin-based adjuvants

Saponins are triterpene glycosides obtained from the bark of the South American soap bark *Quillaja saponaria*. Among several classes of saponins, QS-21 (Matrix-M) is the active purified fraction of *Quillaja saponaria* that has been evaluated for adjuvant properties. QS-21-adjuvanted vaccines activate both T_H_1 and CD8 T-cells, leading to a robust, balanced antibody and cell-mediated responses with minimal reactogenicity (167, 168). Matrix-M™ 40 nm nanoparticles are composed of different fractions of *Quillaja* saponins, phospholipid, and cholesterol. Reimer et al. demonstrated that Matrix-M adjuvant recruits and activate innate and adaptive immune cells, including granulocytes, dendritic cells, and macrophages in vaccine-draining lymph nodes within 48 hours of immunization (169).

Unlike Alum, QS-21 does not promote antigen depot to effect slow release of antigen from injection site (170). However, Marty-Roix et al. reported that QS-21 coupled with MPLA elicits NLRP3-dependent inflammasome activation, leading to the production of IL-1β and IL-18 secretion in both dendritic cells and macrophages *in vitro* (171). IL-1β and IL-18 promote T_H_17 cell maturation or drive INF-γ-mediated T_H_1 responses; inflammasome activation mediated by QS-21 might be critical for development of helper T cells for antibody production. In the same study, however, NLRP3-deficient mice immunized with gp120 proteins adjuvanted with QS-21 showed higher levels of T_H_1 and T_H_2 T-cell responses, and increased IgG1 and IgG2c, indicating that NLRP3-dependent inflammasome may have a negative regulatory role in humoral and cell-mediated immune responses.

Several studies have examined the mechanism of saponin-based adjuvant-aided cross-presentation in human and murine DCs. Initially, studies by Schnurr et al. showed that ISCOMATRIX generates specific class I epitopes of the cancer antigen (NY-ESO-1) by an alternative, proteasome-dependent processing pathway in human DCs (172). The authors also discovered that lysosomal leakage of antigens is driven by lysosomal proton pumps and such antigen translocation is restricted to myeloid DCs. Likewise, Welsby et al. showed that QS-21 promotes the activation and maturation of human monocyte-derived DCs, as indicated by increased levels of IL-6, TNF-α, IL-6, CD86, and HLA-DR (173). Further analysis demonstrated that QS-21 is taken up by human monocyte-derived DCs *via* cholesterol-dependent endocytosis leading to lysosomal destabilization and formation of pores in the lysosomes (174). Such destabilization of lysosomes is critical, as this will allow the exogenous antigens to be released into cytosol for further processing for efficient cross-presentation. The authors have since illustrated that mice deficient in lysosomal proteases (cathepsin B) immunized with QS21-adjuvanted vaccines had fewer antigen-specific CD4 and CD8 T cells (173). Cathepsin B deficiency also negatively affected the polyfunctionality of antigen-specific effector CD4/8 T cells, as measured by their ability to coproduce IFN-γ, IL-2, and TNF-α, suggesting that QS21-mediated adjuvanticity likely requires lysosomes as a part of effective DC cross-presentation.

Using Immune stimulating complexes (ISCOM), which contains QS-21, den Brok et al. demonstrated that saponin-based adjuvant (SBA)-induced cross presentation in murine BMDCs by mechanisms independent of co-stimulatory molecules, CD80 and CD86 (175). Instead, the accumulation of lipid bodies, presumably derived from the adjuvant itself, is associated with enhanced DC cross-presentation by murine monocyte-derived CD11b+ DCs both *in vivo* and *in vitro*, as shown in **Figure 3**. Consistent with this finding, pharmacological inhibition of lipid body formation abrogated saponin-induced antigen cross-presentation both *in vivo* and *in vitro* in mice. Mechanistically, SBA-induced DC-cross-presentation engages cytosolic pathway, as evidenced by enhanced antigen escape from endosomes. However, SBA-induced DC-cross presentation did not entail ROS production nor require NOX2 complex, unlike the conventional cytosolic pathway. Therefore, SBA-mediated antigen escape from endosomes likely does not involve disruption of cellular membranes from ROS-induced damage. Moreover, recent work suggests that SBA-aided cross-presentation requires PERK activation for potentiating CD8 T cell responses, but PERK activation was not required for SBA-mediated LB formation (176). Future studies should elucidate the cellular determinants of SBA-induced LB formation and the functional role of SBA-induced lipid bodies in regulating cytosolic pathway of SBA-mediated DC cross-presentation.

### E) TLR adjuvants

The ligation of TLR ligands to the corresponding receptors induces DC maturation, leading to increased expression of co-stimulatory molecules and production of pro-inflammatory cytokines. The first TLR agonist that was clinically approved for human use as a vaccine adjuvant was Monophosphoryl lipid A (MPL-A), which is a detoxified version of LPS that lacks lipid A. TLR4 agonists are already incorporated into licensed vaccines against human papilloma virus (HPV; Cervarix^®^), hepatitis B virus (HBV; Fendrix^®^, Supervax^®^) and melanoma (Melacine^®^) (177–181). Recent clinical trials on Shingrix also demonstrate the feasibility of MPL as an adjuvant, as evidenced by robust protection against herpes zoster in older adults and efficacy in immunocompromised individuals (182).

Various TLR agonists are currently being used as vaccine adjuvants. For example, AS04 is MPL adsorbed onto aluminum hydroxide or aluminum, which has been known to elicit strong T_H_1-based immunity. MPLA triggers the production of pro-inflammatory cytokines, including IL-6 and TNF-α, through JNK-mTOR-NF-kB signaling pathway (183, 184). MPLA also stimulates DC maturation and suppress immune tolerance by inhibiting regulatory T cells. MPLA also induces T_H_1-skewed immune responses by enhancing IFN-γ production by antigen-specific CD4+ T cells (185, 186). In addition to MPLA, Glucopyranosyl Lipid A (GLA)-SE has been developed, which is synthetic version of LPS. GLA-SE was first evaluated as a vaccine adjuvant against influenza viruses (H5N1) in a phase II clinical trial (187). In this trial, authors learnt that low dose GLA-SE-adjuvanted vaccines induced both humoral and cell-mediated immune responses. The authors also evaluated GLA-SE as a vaccine adjuvant for tuberculosis vaccines; similarly, GLA-SE adjuvant elicits humoral and T_H_1-immune responses in vaccines against TB (188). Together, TLR4 agonists, such as MPLA and GLA-SE, can be used as immune potentiators to elicit potent, balanced CD8 and T_H_1 immune responses to vaccines against infectious diseases.

CpG ODNs are synthetic oligonucleotides composed of at least two unmethylated pairs of cytosine and guanine deoxynucleotides joined by a phosphate-containing linking molecule. These constructs mimic unmethylated DNA fragments from bacteria and viruses, which act as TLR 9 ligands. Like TLR4 agonists, activation of TLR9 by CpG recruits TNF receptor-associated factor 6 (TRAF6), IL-1R associated kinase (IRAK), and MyD88. This subsequently leads to the activation of NF-kB and AP-1, which results in increased secretion of inflammatory cytokines and chemokines (189). CpG has undergone clinical testing in humans and has also completed a phase 3 clinical trial, as an adjuvant in HBV vaccine (Hepislav^®^) (190–194).

Soluble TLR ligands, such as LPS and CpG, have direct impacts on cross-presentation of antigens in DCs, which makes them even ideal candidates for inducing potent T-cell based immunity. Upon DC maturation induced by TLR4 agonists, the processes associated with cross-presentation, such as scavenger receptor-mediated phagocytosis and phagolysosomal fusion, are enhanced within first hours of TLR4 activation, followed by downregulation of antigen internalization and molecular components required for cytosolic delivery of antigen, as illustrated in **Figure 3** (195, 196). Gupta et al. discovered that MHC-I molecules are not derived from the ERGIC upon TLR stimulation, because ERGIC components were recruited to the phagosomes, independent of TLR signaling. However, TLR4 stimulation results in accumulation of MHC class I molecules derived from endocytic recycling compartment (ERC; marked by Rab11a, VAMP3, and VAMP8) to phagosomes. Mechanistically, TLR-mediated MyD88 dependent IKK2 phosphorylation of SNAP23 mediates ERC-phagosome fusion. It was also shown that silencing Rab11a resulted in dissipation of perinuclear reserves of MHC-I and abrogated TLR-mediated cross-presentation. This finding is consistent with the previous finding, as TLR recruitment is not dependent on TLR-mediated signaling (197). Alloatti et al. also showed that activation of TLR4 leads to delayed phagosomal maturation and antigen degradation, thereby inducing the formation of intracellular peri-nuclear clustering of lysosomes mediated by Rab34 (198). These findings collectively suggest that TLR-based adjuvants likely engage vacuolar pathway to potentiate effective CD8 T cell responses. As previously described, the primary source of MHC-I is ERC, which suggests that peptide loading occurs within phagosomes, rather than ER. Additionally, maturation of phagosomes occurs rapidly within first few hours of TLR signaling (199, 200); hence, antigens are likely to be processed within phagosomal proteases, rather than in cytosolic proteasomes.

The TLRs are either located on the plasma membrane (TLRs 1, 2, 4, 5, and 6) or intracellularly (TLRs 3, 7, 8, and 9) within endosomes, but how the location of TLRs dictates efficiency of DC cross-presentation remains controversial. For instance, intracellular TLRs (TLR 3,7, and 9), which are mostly found in endosomes, require internalized ligands such as nucleic acids (RNA and DNA) to activate downstream signaling pathways (MyD88-IRF7 pathway or the MyD88-NFκB pathway). TLR9 that is predominantly expressed by DCs and B cells, can potently respond to CpG, which is known to induce Th1-based responses and potent cytotoxic CD8+ T lymphocytes (90, 201). Therefore, TLR9 agonists were successfully used as in both prophylactic and therapeutic cancer vaccines against melanoma or malignant glioma in mice (202–204). However, mice and humans express different levels of TLR9 in DCs, which leads to reduced inflammatory cytokines and cross-presentation in human DC subsets (205, 206). As a result, it is difficult to use CpG-based vaccines to potentiate anti-tumor immunity, because CpG-mediated T cell response cannot be optimized individually (207). Future studies need to carefully dissect whether or how the location of TLRs can determine the efficacy of cross-presentation in murine and human DC subsets.

### F) Carbomer-based nano-emulsion adjuvant

Carbomers are synthetic high-molecular-weight polyacrylic acids cross-linked with allyl sucrose, which have been used extensively as emulsifiers, gel-forming substances, stabilizers of suspensions, and binders in tablets in pharmaceutical industry (208, 209). Various types of carbomers (e.g. Carbomers-910, -934, -934P, -940, and -941) have low toxicity when ingested and caused no pathological conditions in laboratory animals such as mice and rats. Also, clinical studies with carbomers suggest that they do not cause skin sensitization or irritation.

Several vaccine formulations based on polyacrylic acids such as Carbomers™ or Carbopols™, have been screened for adjuvant activity in mice (210–212). Among them, Adjuplex^®^ (ADJ, Advanced Bioadjuvants) is a carbomer-based nanoemulsion adjuvant (CBA) that consists of biodegradable matrix of carbomer and purified soybean lecithin formulated as submicron-sized liposomes (nanoliposomes). As a vaccine adjuvant, ADJ has several advantages. First, purified lecithin, a major component in ADJ, is often utilized as an emulsifier, antioxidant, or stabilizer (213, 214). Because of its unique biochemical properties, ADJ presumably facilitates the formation of lecithin-derived liposomes, which improves antigen delivery to appropriate cellular compartment in DCs (215, 216). Also, ADJ is a versatile vaccine adjuvant that can be used with diverse macromolecules, including DNA, proteins, and polysaccharides. Further, ADJ can be administered by different routes such as subcutaneous, intranasal, and intramuscular routes (217–223). Lastly, ADJ does not contain detergents, oil, preservatives, and substances of animal- or microbial-origin, which reduces the reactogenicity of vaccine formulation. As a vaccine adjuvant, ADJ has been tested extensively using various antigens, such as Hepatitis C, rotavirus, HIV, fungal antigens, and influenza viruses in different animal models, including mice, rats, rabbits, pigs, goats, and nonhuman primates for stimulating balanced and robust adaptive immune responses (224–229). For instance, in mice, carbomer-based adjuvant vaccines have potent immune activating properties and elicits protective adaptive immunity against influenza challenge by eliciting balanced T_H_1 and antibody responses (230). Also, glycoproteins of HIV proteins or deglycosylated HIV-Env trimers or cleavage-independent HIV-1 trimers formulated with ADJ elicited strong neutralizing titers of antibodies compared to Alum (231). Carbomer-based adjuvants also have been used in combination with MF59 to enhance antibody responses to HIV-1 envelope glycoprotein (210). Collectively, ADJ is a non-toxic, immunomodulatory adjuvant that can improve vaccine potency, particularly by enhancing antigen delivery and inducing strong CD8 and T_H_1 responses. Based on the promise seen in studies with laboratory animals, ADJ is now in phase I human clinical trials for cocaine vaccines (232, 233).

For past years, our group and others have extensively tested the ability of ADJ to stimulate potent and durable CD4 and CD8 T-cell based immunity to viral and intracellular bacterial infections. An initial report showed that ADJ-containing subunit vaccine induced humoral immunity and protected against influenza virus in mice (230). Subsequently, Gasper et al. reported that mice mucosally immunized with subunit antigens formulated in ADJ generated potent antigen-specific CD8 T cell response in the lungs and airways, which engendered protective immunity to influenza A viruses (217). Interestingly, subcutaneous and intranasal vaccination generated systemic and mucosal T cell memory respectively, but only mucosal T cell memory elicited by intranasal vaccination protected against influenza virus. Other studies from our group demonstrated that ADJ robustly stimulates systemic antigen-specific CD4 and CD8 T-cell responses to subunit protein antigen, and protected against vaccinia virus, *Listeria monocytogenes (L. monocytogenes*) and respiratory fungal infections (221, 223, 234). Specifically, upon subcutaneous vaccination of mice, ADJ elicited effector CD8 T cells that differentiated into a distinct subset of granzyme B-expressing CD27^LO^ ‘effector-like’ memory CD8 T cells, which provided highly effective immunity to intracellular bacteria *L. monocytogenes* in spleen and liver (222). Additionally, we have reported that ADJ, in combination with TLR agonists CpG and GLA, stimulated high numbers of tissue resident memory CD4 and CD8 T cells in the respiratory tract and protected against antigenically distinct strains of influenza viruses (218–220). Recently, Kingstad-Bakke et al. leveraged this ADJ-based adjuvant platform to develop broadly protective T-cell based vaccines against SARS-CoV-2 (235). In this recent study, using a spike protein-based subunit vaccine strategy that elicits potent T-cell-based immunity in lungs and spleen, authors demonstrated that both mucosal and parenteral vaccination provide effective protection against pulmonary challenge with the homologous strain of SARS-CoV-2. Strikingly, systemic or mucosal T cell memory to the spike protein of the original SARS-CoV-2 protected against the B.1.351 β variant of SARS-CoV-2, in the absence of detectable neutralizing antibodies.

Like vaccine adjuvants, various innate immune cells, including monocytes, neutrophils, and conventional dendritic cells, are rapidly recruited within 24-48 hours of intradermal, intraperitoneal, and intranasal vaccination of ADJ-containing vaccines at the injection sites (219, 222, 230). CD8 T cell response induced by mucosal or parenteral administration of vaccine antigens formulated in ADJ was ablated in BATF-3-deficient mice (220, 222). These data suggest that stimulation of CD8 T cell responses to subunit vaccine antigens formulated in ADJ requires cross-presentation, presumably by BATF3-dependent conventional migratory DCs. Following intradermal and intranasal administration of ADJ-based vaccines, there were increased numbers of antigen-containing monocytes in DLNs of vaccinated mice. However, unexpectedly, we found that impaired accumulation of monocytes induced by CCR2 deficiency did not significantly affect the activation and expansion of antigen-specific CD8 T cells in spleens or lungs. These findings suggest that monocytes are not required for ADJ-driven antigen cross-presentation and/or for driving the accumulation of antigen-specific CD8 T cells *in vivo* (220, 222). Interestingly however, loss of pulmonary monocytes in CCR2^-/-^ mice led to substantive increase in the total numbers of tissue-resident memory CD8 T cells in lungs of vaccinated mice (220). Hence, pulmonary monocytes appear to have a negative regulatory role in driving mucosal imprinting and development of lung-resident CD8 T cells induced by ADJ-based vaccine. More mechanistic studies are warranted to determine whether or how other innate immune cells, such as neutrophils, can dictate the functionalities and the formation of lung-resident CD8 T cells afforded by ADJ in the lung.

ADJ also induces moderate NLRP3-dependent inflammasome activation in DCs *in vitro*, as indicated by increased IL-1β and IL-18 production in ADJ-treated DCs (221). In particular, addition of TLR-agonist (GLA) in conjunction with ADJ, induced a strong IL-1β response in DCs, suggesting that combination adjuvants appear to trigger potent inflammasome activation in DCs (218). ADJ also triggers potent inflammasome activation *in vivo*, because high levels of IL-1β were detected in lungs of mice intranasally vaccinated with ADJ+GLA within 24-48 hours (219). These collectively suggest that ADJ-containing vaccines likely induce inflammasome activation, regardless of the route of vaccination. However, the biological significance of NLRP3-dependent inflammasome activation by ADJ is unknown. For example, caspase-1-deficient mice were still able to generate antibodies when they were immunized with vaccines that contain Carbopol (a polyanionic carbomer) as an adjuvant (236). Lee et al. also demonstrated that ADJ-mediated DC-cross presentation was unaffected by DCs deficient in NLRP3-dependent inflammasome activation both *in vitro* and *in vivo*, suggesting that ADJ-mediated DC cross-presentation is independent of NLRP3-dependent inflammasome activation (221). How carbomer-based adjuvants trigger inflammasome activation remains elusive, because phago-lysosomal destabilization after adjuvant phagocytosis, such as Alum and Carbopol, is an important step in inflammasome activation (237). While the authors did not directly interrogate whether or how ADJ is taken up by DCs, Lee et al. demonstrated that ADJ increased lysosomal pH, which in turn may result in lysosomal stabilization. Future studies are warranted to determine how and whether ADJ triggers inflammasome activation in DCs and the role of inflammasome activation in engendering protective cell-mediated immunity *in vivo.*

To understand the molecular basis for how carbomer-based adjuvants potentiate CD8 T cell-based immunity, Gasper et al. initially examined whether ADJ affected antigen processing and the ability of ADJ-treated DCs to activate naïve CD8 T cells *in vitro*. The authors found that ADJ alters antigen processing and intracellular localization of antigens in DC 2.4 cells (immature DC-like cell lines), leading to robust proliferation of OT-I CD8 T cells (217). Later, Lee et al. showed that ADJ-mediated cross-presentation entailed ROS-dependent mechanisms of endosomal alkalization and antigen escape to cytosol, proteasomal antigen degradation and TAP-facilitated loading of MHC I molecules, as illustrated in **Figure 3** (221). Typically, upon ligation of TLR, DCs rapidly engage both oxidative phosphorylation and aerobic glycolysis to support the anabolic demands required for expansion of Golgi apparatus and ER for *de novo* fatty acid synthesis, and production of inflammatory and anti-inflammatory cytokines (238, 239). Unlike this paradigm, ADJ-mediated DC cross-presentation occurs in a unique metabolic state, which is characterized by basal levels of glycolysis and profound disengagement of mitochondrial respiration. Lipidomics of ADJ-treated DCs also suggest substantive alterations in cellular lipid composition; pharmacological inhibition of lipid body formation markedly abrogated ADJ-aided DC cross-presentation. Hence, carbomer-based adjuvant aided DC cross-presentation entails endosomes-to-cytosol pathway for mounting effective CD8 T cell responses under a relatively low cellular metabolic state. Further studies are needed to examine how the formation of lipid bodies, or a low metabolic state affects different steps of ADJ-mediated DC cross-presentation, such as antigen leakage from endosomes, MHC-I recycling, and antigen degradation. During cytosolic pathway of cross presentation, cytosolic antigens are degraded into peptides by proteasomes and such peptides traffic into ER or endosomes by mechanism(s) that are dependent upon transporters associated with antigen processing (TAP). To reiterate, the past studies found that TAP1 deficiency abrogated ADJ-mediated cross presentation, but further studies will be needed to determine whether TAP1-dependent MHC I peptide loading occurs in ER or phagosomes in ADJ-stimulated DCs.

## Concluding remarks

As discussed in this review, enhancing the efficiency of dendritic cell cross-presentation is a crucial bottleneck to developing prophylactic and therapeutic vaccines that can elicit potent CD8 T cell responses. Cross-presentation is an inherently complicated process but consists of elaborate series of molecular steps that can be targeted for potentiating CD8 T cell responses *via* generation of alkaline phagosomal environment, promoting antigen escape from phagosomes, and recruitment of ER components to the phagosomes. Whether many of the current proposed mechanisms relevant to cross-presentation are critical for cross-priming *in vivo* remains elusive. It is also unknown whether cytosolic or vacuolar pathway is utilized by distinct DC subtypes in different lymphoid or non-lymphoid organs. Although our current model suggests dichotomous pathways of cross-presentation (i.e. cytosolic versus vacuolar), both pathways are likely functional *in vivo* (240). Indeed, it will likely be advantageous to engage both pathways to potentiate effective CD8 T cell responses by harnessing different adjuvants that can engage different modes of cross-presentation. Some adjuvants, including saponin-based and carbomer-based adjuvants, induce intracellular lipid body formations, which are critical for DC cross-presentation. However, how intracellular LBs affect different steps of DC cross-presentation remain unknown. There is a recent growing interest in engaging DC cross-presentation as a therapeutic cancer vaccine (241–243); various aspects of anabolic and catabolic processes have emerged as crucial factors that control DC effector functions, including DC activation and migration (238, 239, 244–246). Whether or how different steps of metabolic processes modulated by immune adjuvants could affect different facets of DC cross-presentation needs to be further evaluated. Lastly, whether many of these findings can be recapitulated in different human DC subsets is unknown. Therefore, future studies are warranted to mechanistically dissect the pathways of cross-presentation in different human and murine DC subsets and to solve remaining controversies in the context of vaccine adjuvants.

An ideal adjuvant for inducing CTL immunity would target antigen for cross-presentation and maximize the differentiation of memory CD8 T cells. Many adjuvants act as antigen delivery systems (e.g. Alum, MF59, QS-21) and promote DC cross-presentation by augmenting antigen uptake and processing, but perform poorly in enlarging the magnitude of CD8 T cell memory. Concomitant engagement of multiple innate signaling pathways is a prerequisite to programming durable and potent antibody and T cell responses (247), and hence it is likely that targeted cross-presentation need to be partnered with appropriate innate immune signaling to elicit strong and durable CTL immunity. For example, ASO4 (aluminum salt plus monophosphoryl lipid A [MPL; TLR4 agonist]) in the Cervarix human papilloma virus vaccine is a clinically approved vaccine adjuvant that is both potent and safe. In this regard, ADJ is an excellent adjuvant that can enhance DC cross-presentation, but less able to expand the pool of memory CD8 T cells. By combining ADJ with TLR4 or 9 agonists (GLA or CpG), we leveraged the antigen-targeting and mucosal imprinting properties of ADJ and the immune modulatory effects of innate immune signaling to program potent CTL memory in mice. Subunit proteins formulated in the combination adjuvant (ADJ+GLA or ADJ+CpG) and administered intranasally potently elicited tissue-resident memory T cells in the respiratory tract and provided effective and durable defense against antigenically disparate strains of influenza A virus. By contrast, as a component of a parenteral vaccine, ADJ alone was more effective than the combination adjuvant (ADJ with GLA or CpG) in eliciting systemic protective CTL memory to *L. monocytogenes*. These findings suggest that the tenets for eliciting protective CTL memory with adjuvanted subunit vaccines are different for mucosal and parenteral vaccines.

## Author contributions

WL and MS wrote the manuscript. WL made the figure illustration. All authors contributed to the article and approved the submitted version.

## Funding

This work was supported by PHS grant U01 AI124299, R21 AI149793-01A1 and John E. Butler professorship to MS. WL was supported by a predoctoral fellowship from the American Heart Association (18PRE34080150) and dissertation completion fellowship from University of Wisconsin-Madison.

## Acknowledgments

The figure illustrations were made by Biorender.

## Conflict of interest

The authors declare that the research was conducted in the absence of any commercial or financial relationships that could be construed as a potential conflict of interest.

## Publisher’s note

All claims expressed in this article are solely those of the authors and do not necessarily represent those of their affiliated organizations, or those of the publisher, the editors and the reviewers. Any product that may be evaluated in this article, or claim that may be made by its manufacturer, is not guaranteed or endorsed by the publisher.
